# STATE GENEROSITY IN HOME- AND COMMUNITY-BASED SERVICES (HCBS): VARIATIONS AND IMPACT ON DEMENTIA OUTCOMES

**DOI:** 10.1093/geroni/igad104.1498

**Published:** 2023-12-21

**Authors:** Zijing (Flora) Cheng, Yue Li

**Affiliations:** University of Rochester Medical Center, Rochester, New York, United States; University of Rochester Medical Center, Rochester, New York, United States

## Abstract

To meet the needs of community-living older adults and reduce overall medical spending, home- and community-based services (HCBS) provide assistance and care for persons with significant physical and cognitive limitations. Together, Medicaid long-term services and supports (LTSS) coverage and the Title III program under the Older Americans Act (OAA) of 1965 support the majority of the HCBS for older adults living in the U.S. with low-income and/or disabilities. We evaluated state variations in generosity of supporting HCBS in recent years and how these variations affect the healthcare outcomes of community-living older adults and socio-economic and racial disparities in dementia outcomes. State HCBS generosity from the perspectives of program participation and intensity of services was defined by the exploratory and the confirmatory factor analysis with data from multiple sources, including state reports from the National Association of State Budget Officers. State data was linked to a nationally representative sample of older adults who enrolled in the Health and Retirement Study in 2020. Analyses revealed that overall state supports for HCBS increased over time in recent years while state Medicaid expenditures for institutional long-term care remained largely stable. State supports through both Medicaid and the OAA programs for HCBS varied substantially and persisted in recent years. Discussion will focus on instrumental variable analyses that determine the causal impact of state variations in HCBS generosity on health outcomes (i.e., unmet need; hospitalization) for persons living with dementia persons.

